# Lyme Borreliosis Awareness, Risk Perception, and Willingness to Vaccinate in France: Where Do We Stand?

**DOI:** 10.3390/pathogens15101050

**Published:** 2026-10-02

**Authors:** Cyrille Pouyaban, Hannah Gould, Andreas Pilz, Jennifer Moisi, Charles Nuttens

**Affiliations:** 1 Primary Care Medical Affairs, Pfizer France, 75014 Paris, France; 2 Global Vaccines Medical Affairs, Pfizer, New York, NY 10001, USA; 3 Global Vaccines Medical Affairs, Pfizer, 2304 Vienna, Austria; 4 Global Vaccines Medical Affairs, Pfizer, 75014 Paris, France

**Keywords:** Lyme borreliosis, tick, population survey, awareness, risk perception, willingness to vaccinate

## Abstract

Lyme borreliosis (LB) is the most common tick-borne disease in the Northern Hemisphere. In France, despite rising LB incidence, preventive measures remain limited and mainly rely on personal protective behaviors. As efforts advance toward developing an LB vaccine, it is crucial to gain a deeper understanding of public awareness, preventive practices, and vaccine acceptance. A cross-sectional online survey of French adults aged 18–65 years was conducted between November 2022 and January 2023 to collect data on sociodemographic characteristics, outdoor activity patterns, tick and LB awareness, perceived risk, preventive behaviors, and willingness to vaccinate. Among the 1803 eligible respondents, tick awareness was high (94.1%), with LB awareness slightly lower (74.5%). Among tick- and LB-aware respondents, 39.4% and 29.7% perceived themselves to be at high risk of being bitten by a tick and of contracting LB, respectively. The most common preventive behaviors were post-exposure tick checks (39.8%) and wearing protective clothing (38.5%). Concern about side effects (63.2%) and cost (42.4%) were the main barriers to vaccination. Willingness to vaccinate reached 68.3% in a hypothetical scenario in which the vaccine was both reimbursed and recommended by a healthcare professional. Our findings suggest that willingness to vaccinate against LB could be high if a future vaccine were reimbursed and supported by healthcare professionals.

## 1. Introduction

Lyme borreliosis (LB) is a tick-borne disease caused by bacteria belonging to the *Borrelia burgdorferi sensu lato* complex. It is the most common tick-borne disease in the Northern Hemisphere [1]. In Europe, an average of 132,000 LB cases is reported to surveillance systems annually, with the highest incidence rates observed in Finland, Estonia, Lithuania, Poland, Slovenia, Switzerland, and France [2]. In France, epidemiological surveillance primarily relies on the national sentinel network of general practitioners (Réseau Sentinelles) to estimate the number of cases in the primary care setting, and the national hospital database (Programme de Médicalisation du Système d’Information) to identify hospitalized cases. Between 2010 and 2024, the annual incidence rate of LB rose from 42 to 53 cases per 100,000 inhabitants, with a peak observed in 2018 at 104 cases/100,000 population [3]. The annual hospitalization rate ranged from 1.2 to 1.5 per 100,000 inhabitants since 2009, before declining and stabilizing below 1.0 per 100,000 between 2021 and 2024 (0.96/100,000 in 2024) [4].

Treatment of LB is primarily based on curative antibiotic therapy, including tetracyclines (e.g., doxycycline), β-lactams (e.g., amoxicillin or ceftriaxone), and macrolides (e.g., azithromycin) [5]. Prevention of LB primarily relies on the adoption of personal protective measures such as performing tick checks, wearing protective clothing, and using insect repellent; however, these methods have varying levels of effectiveness due to low uptake or poor adherence [6]. Community-level interventions include host-targeted infection control, tick habitat modification, and biological or chemical infection control. Public health education, through widespread awareness campaigns, also plays a critical role in reducing exposure risk. No licensed LB vaccine is currently available, though several vaccine candidates are under development [7].

Recent international studies have investigated knowledge, attitudes, and behaviors related to LB, as well as assessed the acceptability of a potential LB vaccine [8,9,10]. These studies are needed to inform public health stakeholders and support the development of national strategies for LB prevention and control. In France, two population surveys assessed the impact of the National Lyme Plan, launched in 2016, against tick-borne diseases [11]. The surveys, conducted by Santé Publique France (SPF) before and after (in 2016 [12] and 2019 [13], respectively) National Lyme Plan implementation showed an increase in LB awareness and protective behaviors between 2016 and 2019. Since then, the COVID-19 pandemic has influenced public perceptions, both in terms of individual and collective approaches to health, and has contributed to a growing mistrust in vaccination [14].

This article evaluated knowledge, attitudes, and behaviors related to LB and assessed willingness to vaccinate against LB in the French population. While European-level results of this multinational survey have been reported previously [8,15], the present study provides the first in-depth, France-specific analysis of these data, including estimates stratified by region, age group, gender, and urbanicity, and is, to our knowledge, the first assessment of willingness to vaccinate against LB in France.

## 2. Materials and Methods

### 2.1. Survey Design

A cross-sectional online survey involving adult participants (aged 18–65 years) from 20 European countries was conducted between November 2022 and January 2023. The survey took approximately 15 min to complete and assessed participants’ awareness of ticks and LB, their personal history of tick bites and LB diagnosis, risk perception, as well as their attitudes towards vaccination. It also gathered information on demographic variables such as household income, gender, age, urbanicity, dog ownership, and time spent outdoors between 1 April and 30 November. To ensure data integrity, completed surveys underwent quality control procedures to eliminate entries that showed signs of rushed completion or random responding. Further methodological details, including how sample sizes were determined and allocated across countries, the representativeness of the sample, the procedures used to calculate sampling weights, and an English version of the full survey instrument are available in Gould et al. [8]. Briefly, participants were recruited from a pre-existing online research panel maintained by Ipsos and received routine panel-based compensation for survey completion, in accordance with the panel provider’s standard policies.

Prior to completing the survey, participants were informed of its voluntary nature, their right to withdraw at any time, the type of data being collected, and the relevant data privacy policies. Confirmation of understanding and consent was gathered via designated checkboxes before proceeding.

### 2.2. Analysis

This manuscript analyzed data exclusively from French respondents. Data were weighted by geographic region, gender, and age to reflect their distributions in the French adult population aged 18–65 years. Respondents were categorized into four age brackets (aged 18–29, 30–39, 40–49, and 50–65 years), four gender categories (“women”, “men”, “other”, and “prefer not to answer”), and three urbanicity levels (urban, suburban, and rural). Urbanicity was determined based on self-reported living environment: city (urban), city suburbs or peripheral residential areas (suburban), or countryside (rural).

The survey design ensured that individuals unfamiliar with ticks or LB were not presented with follow-up questions on these topics. Consequently, denominators differ across survey items: questions on tick bites, tick-related risk perception, and preventive behaviors were asked only of tick-aware respondents, whereas questions on LB history, LB risk perception, and willingness to vaccinate were asked only of respondents aware of both ticks and LB. All survey items were mandatory unless omitted due to programmed skip logic.

All analyses were descriptive. Weighted proportions with 95% confidence intervals (CIs) were computed for categorical variables, and weighted means with standard deviations for continuous variables, overall and stratified by urbanicity, age group, and gender. No formal hypothesis testing or multivariable modeling was performed, in line with the descriptive design of the study. Thus, between-group differences should be interpreted as exploratory.

Of note, this work builds upon previously generated and analyzed data [8,15].

## 3. Results

### 3.1. Participant Characteristics

Among the 24,791 French residents who were invited to participate in the survey, 1868 completed the survey. Following quality control, 1803/24,791 (7.3%) surveys were retained for analysis. Of the 1803 respondents, 94.1% reported awareness of ticks and were asked the tick-related questions, and 74.5% were aware of both ticks and LB and were asked the LB-related questions (weighted proportions).

Participants were evenly distributed by gender and age. Regarding urbanicity, 807/1803 (44.8%) self-reported living in urban areas, 379 (21.0%) in suburban areas, and 617 (34.2%) in rural areas (Table 1).

On average, respondents reported spending 9.7 h per week on outdoor activities away from home (e.g., birdwatching, hiking, walking, running, biking, picnicking, grilling, eating outdoors, or walking a dog) and 8 h per week on outdoor activities at home (e.g., gardening, lawn mowing, reading/sunbathing) between April and November 2022, corresponding to an average of 2.9 and 2.6 outdoor activities per week, respectively. Respondents living in rural areas reported spending slightly more time outdoors than those in other urbanicities (Table 1).

### 3.2. Awareness of Ticks and Lyme Borreliosis

Overall, 94.1% (95% CI: 92.9–95.1) of respondents reported having heard of ticks, and 79.2% (95% CI: 77.2–81.1) of those reported having heard of LB (74.5% of the total number of respondents). Awareness varied by region, ranging from 90% (95% CI: 79.2–95.5) in Centre to 100% (95% CI: 100.0–100.0) in Limousin for ticks, and from 69% (95% CI: 53.8–81.7) in Champagne-Ardenne to 93% (95% CI: 75.7–98.4) in Limousin for LB (Figure 1, Table S1).

Awareness of both ticks and LB increased with age, from 87.2% (95% CI: 83.6–90.1) and 65.3% (95% CI: 60.0–70.2), respectively, among individuals aged 18–29 years to 97.0% (95% CI: 95.3–98.1) and 87.2% (95% CI: 84.1–89.7), respectively, among those aged 50–65 years. Awareness also varied by urbanicity. Awareness of ticks was reported by 91.7% (95% CI: 89.5–93.4) of urban residents, 92.1% (95% CI: 88.9–94.4) of suburban residents, and 98.4% (95% CI: 97.0–99.1) of rural residents. Awareness of LB followed a similar trend: 72.7% (95% CI: 69.3–75.9), 79.9% (95% CI: 75.3–83.8), and 86.6% (95% CI: 83.5–89.1), respectively (Table S1).

### 3.3. Tick Bite and LB History

Among those aware of ticks, a total of 41.0% (95% CI: 38.7–43.4) of respondents reported having been bitten by a tick at least once in their lifetime. The prevalence of tick bites varied by urbanicity, from 35.1% (95% CI: 30.3–40.3) to 47.3% (95% CI: 43.4–51.4) in suburban and rural areas, respectively. Among those aware of ticks and LB, 7.6% (95% CI: 6.3–9.1) of participants reported having received a diagnosis of LB in their lifetime (Table S1), representing 5.7% of all respondents.

### 3.4. Perception of Ticks and LB

Overall, 39.4% (95% CI: 37.1–41.8) and 29.7% (95% CI: 27.3–32.3) of respondents perceived themselves to be at high or very high risk of being bitten by a tick and contracting LB, respectively. Moreover, 83.2% (95% CI: 81.1–85.1) of respondents considered LB as a severe condition and 46.0% (95% CI: 43.3–48.8) were concerned about contracting it (Table 2, Table S2). Perceived risk of tick bites did not vary greatly by urbanicity (rural: 43.5%, 95% CI: 39.5–47.5; suburban: 36.9%; 95% CI: 31.9–42.1; urban: 37.2%, 95% CI: 33.9–40.8). Perceived risk of contracting LB was also similar for residents of rural (29.7%, 95% CI: 25.8–33.8), suburban (25.7%, 95% CI: 20.8–31.2), and urban (31.9%, 95% CI: 28.0–36.1) areas. Adults aged 30–39 years reported a higher perceived risk of both tick bites 47.1% (95% CI: 42.0–52.3) and LB 38.7% (95% CI: 33.0–44.7) than those aged 18–29 (31.3%, 95% CI: 26.7–36.3 and 25.6%, 95% CI: 20.3–31.8, respectively) or 50–65 (37.3%, 95% CI: 33.4–41.3 and 23.1%, 95% CI: 19.6–27.1, respectively) years. Respondents aged 50–65 years had a lower perceived risk of LB (23.1%, 95% CI: 19.6–27.1) and a lower concern about contracting LB (39.9%, 95% CI: 35.6–44.3) than the other age groups, despite having the highest perceived disease severity (85.5%, 95% CI: 82.1–88.4). Women and men had a similar perceived risk of tick bites (41.6%, 95% CI: 38.3–44.9 and 37.1%, 95% CI: 33.8–40.4, respectively) and of contracting LB (28.4%, 95% CI: 25.3–31.9 and 31.2%, 95% CI: 27.6–35.1, respectively) (Table 2, Table S2).

### 3.5. Preventive Measures

Most participants who were tick- and/or LB-aware reported “often” or “always” engaging in the following preventive behaviors: checking for ticks after outdoor exposure (39.8%, 95% CI: 37.5–42.1), wearing protective clothing (e.g., long socks or trousers tucked into socks) (38.5%, 95% CI: 36.2–40.8), avoiding tick-infested areas (35.7%, 95% CI: 33.4–38.0), and using insect repellents such as DEET (18.0%, 95% CI: 16.2–19.9) (Table 3, Table S3).

A higher proportion of women than men reported conducting tick checks and avoiding tick-infested areas. Reported use of insect repellents was higher among younger participants (aged <50 years) than among older adults (aged 50–65 years), for whom the most frequently reported prevention measure was wearing protective clothing. The proportion of respondents reporting tick checks was higher among rural residents than among urban and suburban residents (Table 3, Table S3).

### 3.6. Willingness to Vaccinate

Factors positively influencing willingness to vaccinate included healthcare professional (HCP) recommendation (63.4% [95% CI: 60.7–66.0] reported “4” or “5” on a scale from 1 [strongly disagree] to 5 [strongly agree] to receive the vaccine if it was recommended by an HCP), official health authority guidelines (52.3%, 95% CI: 49.5–55.0), friend and family recommendation (45.1%, 95% CI: 42.4–47.9), workplace offering vaccination (40.0%, 95% CI: 37.3–42.7), and social media recommendation (24.8%, 95% CI: 22.5–27.2) (Table 4, Table S4). Willingness to vaccinate due to social media or friend and family recommendations was more pronounced among young compared to older adults (35.9% [95% CI: 29.8–42.5] and 57.8% [95% CI: 51.1–64.2], respectively, for those aged 18–29 years vs. 13.9%, 95% CI: 11.1–17.3 and 38.4%, 95% CI: 34.2–42.8, respectively, for those aged 50–65 years) and among urban residents compared to rural residents (34.0% [95% CI: 30.0–38.2] and 52.6% [95% CI: 48.3–57.0] vs. 18.4% [95% CI: 15.2–22.0] and 38.7% [95% CI: 34.5–43.0], respectively). HCP recommendation was the highest social factor across all urbanicity, age group, and gender categories (Table 4, Table S4).

The primary barrier to vaccination was fear of side effects, with 63.2% (95% CI: 60.5–65.8) of respondents having concerns about the possible side effects of the vaccine; this proportion was higher among women (69.5%, 95% CI: 66.1–72.9) than men (55.5%, 95% CI: 51.4–59.5). The cost of vaccination, when not reimbursed, was a concern for 42.4% (95% CI: 39.7–45.1) of respondents, particularly younger adults (48.5% [95% CI: 42.0–55.2] for those aged 18–29 years vs. 34.8% [95% CI: 30.7–39.1] for those aged 50–65 years).

Overall, 68.3% (95% CI: 65.7–70.8) of respondents reported being likely or very likely to accept vaccination in a hypothetical scenario in which the vaccine was both reimbursed and recommended by an HCP. Additionally, 53.6% (95% CI: 50.9–56.4) of participants stated they would accept vaccination even if multiple doses were required (Table 4, Table S4).

## 4. Discussion

This study was the first to evaluate acceptance of an LB vaccine in France, in addition to updating the awareness and risk perception findings previously gathered by SPF in 2016 and 2019 [12,13]. Conducting this research was important given that: (i) LB is the most common vector-borne disease in Europe, yet the incidence, and therefore the burden, of LB in France is likely underestimated [16], (ii) LB incidence has shown an upward trend in France in recent years [4], and future climate change is expected to considerably influence its epidemiology [17,18], and (iii) Vaccination may soon be added to the arsenal of LB prevention measures, with the most advanced candidate LB6V (VLA15), a hexavalent vaccine codeveloped by Pfizer and Valneva, having recently completed Phase 3 clinical trials [19].

The findings from this study are consistent with those of previous research. For instance, the 2019 survey conducted by SPF revealed a similar level of LB awareness as reported in our study (79% vs. 74.5%). However, the SPF study showed slightly lower proportions of individuals who perceived themselves to be at high risk of being bitten by a tick (24.9% vs. 37.1%) or having experienced a tick bite (30.2% vs. 38.6%) compared to our results [13]. These findings reflect a generally sound understanding of LB in the French population. The relatively high proportion of participants (5.7% of total) reporting a prior diagnosis of LB during their lifetime in our study, along with its distribution across age groups and urbanicity, may indicate a self-overdiagnosis in some populations. This contrasts with French incidence data [4,20], which, despite likely being underestimated [16], are based on robust diagnostic criteria for LB, including the presence of erythema migrans or suggestive neurological, articular, cutaneous, or cardiac manifestations in patients with positive serology (i.e., a positive ELISA test confirmed by Western blotting) [3]. The literature has documented this bias, which may stem from misdiagnoses made by HCPs or by the patients themselves [21,22,23]. This could be explained by the fact that erythema migrans diagnosis relies solely on clinical presentation, or by the limited reliability of self-testing kits available in pharmacies [5].

Several points emerge from this study regarding knowledge, perceptions, and behaviors related to ticks and LB. Participants, regardless of their residential setting (urban, suburban, or rural), reported spending considerable time outdoors and described broadly comparable histories of tick bites. In addition, awareness of ticks and LB did not appear to be systematically aligned with local epidemiological patterns or with residence in high-incidence regions. Consistent with findings of Mathews-Martin et al. [24], our results suggest that awareness and risk communication efforts may need to extend beyond rural populations or areas with higher reported LB incidence.

When interpreting these findings, it is also useful to distinguish between hazard (the density of infected *Ixodes ricinus* ticks in the environment), exposure (the frequency and nature of contact with tick habitats), and perceived risk (respondents’ subjective assessment of their own risk). Self-reported outdoor exposure was substantial and broadly comparable across urbanicity levels in our sample. Questing *I. ricinus* ticks have been documented in urban and peri-urban green spaces in France [24], which may partly explain why perceived risk did not differ markedly between urban and rural residents despite differences in awareness. Nevertheless, perceived risk cannot be considered a proxy for actual exposure, as neither tick density nor objectively measured exposure was assessed in this survey.

In terms of preventive measures, and in line with the findings of Septfons et al. [13], the use of repellents remains the least favored option by the public, whereas wearing protective clothing and performing post-exposure body checks are the most commonly adopted strategies. Rural respondents tended to report higher awareness of ticks/LB and use of preventive measures than urban respondents, a finding that may reinforce the need for broad, population-wide communication and information strategies.

Regarding vaccination, the main barrier to uptake lies in concerns about safety. Thus, it appears crucial to avoid repeating the scenario associated with the first LB vaccine LYMErix^®^, introduced in 1998. Though LYMErix^®^ was demonstrated to be effective and initially well-received, it was later the focus of safety concerns related to reported cases of arthritis. This effect was hypothesized to result from sequence homology between OspA and hLFA-1, which is absent from the Valneva-Pfizer product. Additionally, subsequent investigations conducted by the US Food and Drug Administration and the Vaccine Adverse Events Reporting System did not confirm a causal association between vaccination and arthritis [7]. Extensive media coverage, actions by anti-vaccine groups, and class-action lawsuits eventually caused the manufacturer to withdraw the vaccine in 2002 [25]. This precedent warrants careful consideration, especially in a country like France, where a high level of vaccine safety skepticism (45.2%) has been reported [26]. Addressing this concern should therefore be a priority for public health authorities as LB vaccines move closer to public availability [19,27].

More than half of the respondents in our study indicated willingness to receive a multidose vaccine. Of note, the corresponding survey item referred to a vaccine requiring multiple doses, without specifying the number of doses or the potential need for annual boosters; responses may therefore primarily reflect acceptance of an initial multidose series rather than of long-term, repeated revaccination, and may overestimate adherence to the full anticipated schedule. This point will be important to monitor in real-world settings as the most advanced LB vaccine candidate, if licensed, is expected to require a multidose administration schedule (initially given at months 0, 2, 5–9, and 18, followed by annual boosters), making anticipation and careful planning of vaccination campaigns essential. Adherence may also present a challenge, as existing benchmarks for vaccine series requiring at least three doses indicate decreasing adherence with increasing number of doses. In a UK study evaluating hepatitis B vaccination compliance, only 46% and 22% of individuals adhered to the schedules for the second and third doses, respectively [28]. Similarly, a tick-borne encephalitis vaccination study involving 20 European countries showed that the average vaccination adherence in endemic countries was only 21% for the primary series (comprising the first three doses) and dropped further to 7% for the first booster dose (i.e., fourth dose) [29]. In our survey, 68.3% of respondents were in favor of receiving LB vaccination if it was reimbursed and recommended by an HCP. This figure roughly aligns with findings from other studies, in which the majority of participants were in favor of receiving the LB vaccine (75% in Schotthoefer et al. [30], 84% in Niesobecki et al. [10]).

Should an LB vaccine be licensed and recommended, implementing targeted medical education programs would be important to support informed vaccination decisions and vaccine uptake, as healthcare providers play a key role in shaping patient vaccination decisions. However, vaccine hesitancy among French HCPs represents a notable barrier. A comprehensive review revealed that 11% of general practitioners, 20% of medical students, and 44% of nurses reported vaccine hesitancy [31]. Moreover, 16% and 43% of French GPs reported “never” or “sometimes” recommending certain vaccines to patients [32]. Since HCPs must have in-depth vaccine knowledge to effectively communicate their importance to patients, maintaining and potentially strengthening information campaigns targeting both HCPs and the general public (in all urbanicities) will be essential. These efforts should also embrace more innovative information dissemination approaches, particularly to better reach younger and urban populations, for whom traditional channels of communication may prove less effective. This is especially relevant given that a substantial proportion of the population travels to regions with higher LB incidence during the spring and summer, where they may become exposed to the disease through recreational or occupational activities.

This study had several limitations. First, the use of self-reported data from a pre-existing survey panel introduces the potential for selection and recall biases. In particular, only 7.3% of the invited panel members completed the survey and contributed to the analysis. Respondents may therefore differ from non-respondents on unmeasured characteristics, such as interest in ticks and tick-borne diseases or a personal history of symptoms attributed to LB, which may have inflated some estimates, including the self-reported lifetime prevalence of LB. Although quotas and weighting based on gender, age, and regional distribution were applied to enhance representativeness, the possibility of residual confounding cannot be excluded. Second, individuals over the age of 65 years were not included in the survey, as recruitment relied on an online panel in which older adults are under-represented. Because this age group accounts for a substantial proportion of severe LB cases (including neuroborreliosis) in France, our findings, particularly those regarding willingness to vaccinate, cannot be generalized to adults aged >65 years, a population with a potentially high medical need for prevention. Third, the assessment of LB risk perception only involved respondents who indicated prior awareness of ticks and LB. While the majority of participants met these criteria, those lacking awareness were not asked about their risk perception, likely resulting in an underestimation of lower risk perception scores in the overall sample. Finally, all analyses were descriptive; the study was neither designed nor powered to identify independent predictors of awareness, preventive behaviors, or willingness to vaccinate. As such, observed differences between subgroups may be confounded by other factors and should be interpreted as descriptive.

## 5. Conclusion

In conclusion, this study assessed the knowledge, attitudes, and perceptions of LB in a sample of individuals in France, alongside their willingness to be vaccinated against the disease. Our findings revealed both a high level of awareness of ticks and LB and a substantial perceived risk of tick bites and disease. If licensed and recommended by health authorities, an LB vaccine could complement existing preventive measures, provided that efficacy and safety data adequately address the persistent challenge of vaccine hesitancy in France.

## Figures and Tables

**Figure 1:**
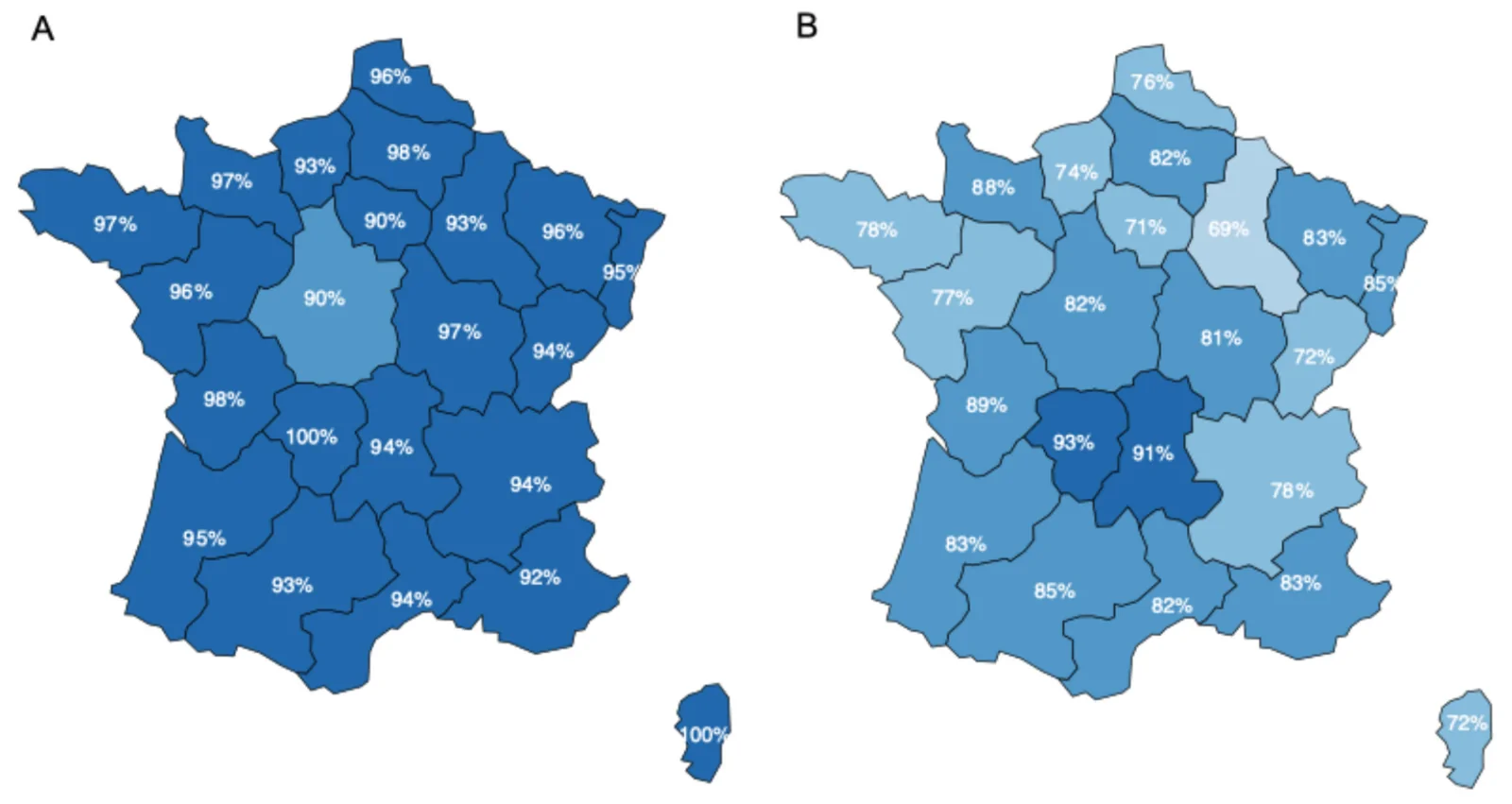
Proportions of tick-aware (**A**) and Lyme-borreliosis-aware (**B**) respondents in each region of France. Lyme borreliosis awareness was measured only for respondents who had indicated tick awareness in the survey.

**Table 1:** Survey respondent demographics (weighted by region, gender, and age) and average weekly number of activities or time spent outdoors from April to November 2022 in France, reported overall and stratified by urban, suburban, and rural urbanicities.

Characteristic	Overall (*n* = 1803)	Urban (*n* = 807)	Suburban (*n* = 379)	Rural (*n* = 617)
Age in years, *n* (%)				
18–29	420 (23.3)	238 (29.5)	75 (19.8)	107 (17.4)
30–39	374 (20.8)	199 (24.7)	69 (18.3)	106 (17.1)
40–49	389 (21.6)	169 (21.0)	78 (20.7)	141 (22.9)
50–65	620 (34.4)	201 (24.9)	156 (41.2)	263 (42.6)
Gender, *n* (%)				
Women	913 (50.6)	387 (48.0)	200 (52.9)	325 (52.7)
Men	885 (49.1)	419 (51.9)	175 (46.3)	291 (47.1)
Other	4 (0.2)	1 (0.1)	2 (0.5)	1 (0.2)
Prefer not to answer	1 (0.1)	0 (0.0)	1 (0.3)	0 (0.0)
Outdoor time away from home *, mean (SD)				
Average reported hours spent outdoors away from home per week	9.7 (8.4)	9.4 (8.0)	8.5 (7.3)	10.7 (9.3)
Average reported number of outdoor activities away from home per week	2.9 (1.4)	2.9 (1.5)	2.7 (1.4)	3.0 (1.4)
Outdoor time at home *, mean (SD)				
Average reported hours spent outdoors at home per week	8.0 (7.1)	7.6 (7.1)	7.4 (6.6)	8.9 (7.3)
Average reported number of outdoor activities at home per week	2.6 (1.3)	2.5 (1.4)	2.6 (1.3)	2.7 (1.2)

* Between 1 April and 30 November. Abbreviations: SD, standard deviation.

**Table 2:** Perceived risk, severity, and concerns surrounding ticks and LB by tick-aware or by tick- and LB-aware respondents who reported “4” or “5” on a scale from 1 (very low) to 5 (very high).

Characteristic	Perceived Risk for Being Bitten by a Tick * % (95% CI)	Perceived Risk for Contracting LB ^$^ % (95% CI)	Perceived Severity of LB ^$^ % (95% CI)	Concern About Contracting LB ^$^ % (95% CI)
**Overall**	39.4 (37.1–41.8)	29.7 (27.3–32.3)	83.2 (81.1–85.1)	46.0 (43.3–48.8)
Urbanicity				
Urban	37.3 (33.9–40.8)	31.9 (28.0–36.1)	81.9 (78.3–85.0)	45.7 (41.4–50.0)
Suburban	36.9 (31.9–42.1)	25.7 (20.8–31.2)	83.2 (78.3–87.2)	44.3 (38.5–50.3)
Rural	43.5 (39.5–47.5)	29.7 (25.8–33.8)	84.4 (81.0–87.4)	47.4 (43.0–51.7)
Age, years				
18–29	31.3 (26.7–36.3)	25.6 (20.3–31.8)	80.0 (74.1–84.8)	47.2 (40.7–53.9)
30–39	47.1 (42.0–52.3)	38.7 (33.0–44.7)	79.6 (74.3–84.1)	51.9 (45.8–57.9)
40–49	43.5 (38.7–48.5)	36.2 (31.1–41.7)	84.8 (80.4–88.4)	50.5 (45.0–56.1)
50–65	37.3 (33.4–41.3)	23.1 (19.6–27.1)	85.5 (82.1–88.4)	39.9 (35.6–44.3)
Gender ^§^				
Women	41.6 (38.3–44.9)	28.5 (25.3–31.9)	84.0 (81.1–86.5)	46.9 (43.2–50.6)
Men	37.1 (33.8–40.4)	31.2 (27.6–35.1)	82.1 (78.8–85.0)	44.9 (40.9–49.0)

* Measured for respondents who reported awareness of ticks. ^$^ Measured for respondents who reported awareness of ticks and Lyme borreliosis. ^§^ ‘Other’ and ‘Prefer not to answer’ were not analyzed due to the limited number of respondents in these categories. Abbreviations: CI, confidence interval; LB, Lyme borreliosis.

**Table 3:** Adoption of listed protective behaviors against tick bites by tick-aware respondents who reported “often” or “always” using tick prevention measures.

Characteristic	Conducting Tick Checks % (95% CI)	Using Protective Clothing % (95% CI)	Avoiding Tick Infested Areas % (95% CI)	Using Insect Repellents % (95% CI)
**Overall**	39.8 (37.5–42.1)	38.5 (36.2–40.8)	35.7 (33.4–38.0)	18.0 (16.2–19.9)
Urbanicity				
Urban	35.7 (32.3–39.3)	36.0 (32.6–39.5)	33.9 (30.6–37.4)	20.7 (18.0–23.8)
Suburban	34.9 (30.1–40.1)	38.0 (33.0–43.2)	34.5 (29.6–39.6)	14.1 (10.8–18.2)
Rural	47.5 (43.5–51.5)	41.7 (37.8–45.7)	38.5 (34.7–42.5)	16.8 (14.0–20.0)
Age, years				
18–29	41.6 (36.6–46.8)	38.8 (33.9–44.0)	38.0 (33.1–43.2)	23.2 (19.1–27.9)
30–39	43.9 (38.9–49.1)	37.7 (32.8–42.8)	41.1 (36.1–46.3)	23.2 (19.1–27.9)
40–49	39.6 (34.8–44.5)	36.1 (31.5–41.0)	34.2 (29.7–39.1)	18.3 (14.8–22.5)
50–65	36.4 (32.5–40.4)	40.2 (36.2–44.2)	31.9 (28.3–35.9)	11.4 (9.1–14.3)
Gender ^§^				
Women	43.7 (40.4–47.0)	40.2 (37.0–43.5)	39.2 (36.0–42.5)	19.1 (16.6–21.8)
Men	35.6 (32.4–39.0)	36.6 (33.3–39.9)	31.7 (28.6–34.9)	16.7 (14.4–19.5)

^§^ ‘Other’ and ‘Prefer not to answer’ were not analyzed due to the limited number of respondents in these categories. Abbreviations: CI, confidence interval.

**Table 4:** Facilitators of and barriers to willingness to receive an LB vaccine, reported by tick- and LB-aware respondents who reported “4” or “5”, on a scale from 1 (strongly disagree) to 5 (strongly agree), that the listed factors would impact their willingness to receive an LB vaccine.

Characteristic	Workplace Offering Vaccination % (95% CI)	HCP Recommendation % (95% CI)	Official Published Guidelines % (95% CI)	Friend and Family Recommendation % (95% CI)	Social Media Recommendation % (95% CI)	Cost % (95% CI)	Side Effect % (95% CI)	Multidose % (95% CI)
**Overall**	40.0 (37.3–42.7)	63.4 (60.7–66.0)	52.3 (49.5–55.0)	45.1 (42.4–47.9)	24.8 (22.5–27.2)	42.4 (39.7–45.1)	63.2 (60.5–65.8)	53.6 (50.9–56.4)
Urbanicity								
Urban	47.6 (43.2–51.9)	66.2 (62.0–70.2)	58.2 (53.8–62.4)	52.6 (48.3–57.0)	34.0 (30.0–38.2)	48.6 (44.3–53.0)	64.5 (60.3–68.6)	56.4 (52.1–60.7)
Suburban	36.3 (30.8–42.2)	61.6 (55.6–67.2)	49.9 (44.0–55.9)	43.1 (37.3–49.1)	19.5 (15.2–24.6)	35.8 (30.3–41.7)	61.2 (55.3–66.9)	53.9 47.9–59.8)
Rural	34.3 (30.3–38.6)	61.5 (57.1–65.6)	47.6 (43.2–52.0)	38.7 (34.5–43.0)	18.4 (15.2–22.0)	39.8 (35.6–44.1)	62.9 (58.6–67.0)	50.7 (46.3–55.1)
Age, years								
18–29	42.7 (36.3–49.4)	62.3 (55.7–68.5)	56.0 (49.3–62.4)	57.8 (51.1–64.2)	35.9 (29.8–42.5)	48.5 (42.0–55.2)	62.4 (55.7–68.6)	52.7 (46.0–59.2)
30–39	47.9 (41.9–53.9)	66.9 (61.0–72.3)	54.6 (48.6–60.5)	51.5 (45.5–57.5)	34.3 (28.8–40.2)	49.1 (43.1–55.2)	67.6 (61.7–73.0)	64.1 (58.2–69.7)
40–49	39.5 (34.3–45.1)	60.2 (54.7–65.5)	49.8 (44.3–55.4)	41.5 (36.2–47.1)	26.6 (22.0–31.8)	45.0 (39.5–50.6)	66.5 (61.1–71.5)	46.5 (41.0–52.0)
50–65	35.0 (30.9–39.3)	63.9 (59.5–68.1)	50.8 (46.4–55.3)	38.4 (34.2–42.8)	13.9 (11.1–17.3)	34.8 (30.7–39.1)	59.3 (54.9–63.6)	53.0 (48.5–57.4)
Gender ^§^								
Women	35.6 (32.1–39.2)	60.7 (57.0–64.2)	50.8 (47.1–54.5)	42.4 (38.8–46.1)	22.3 (19.4–25.5)	41.6 (38.0–45.2)	69.6 (66.1–72.9)	50.7 (47.0–54.4)
Men	45.4 (41.4–49.5)	66.9 (62.9–70.6)	54.2 (50.1–58.2)	48.6 (44.5–52.6)	27.8 (24.3–31.5)	43.5 (39.5–47.6)	55.5 (51.4–59.5)	57.2 (53.1–61.2)

^§^ ‘Other’ and ‘Prefer not to answer’ were not analyzed due to the limited number of respondents in these categories. Abbreviations: CI, confidence interval; HCP, healthcare professional.

## Data Availability

The datasets generated during the current study are not publicly available due to the sensitive nature of the survey data and the terms of participant consent, which did not include provisions for public data sharing, but are available from the corresponding author upon reasonable request.

## Supplementary Materials

The following supporting information can be downloaded at: https://www.mdpi.com/article/10.3390/pathogens15101050/s1, Table S1: Awareness of ticks and Lyme borreliosis, and past experience of tick bites and Lyme borreliosis, reported overall and stratified by urbanicity, age group, and gender; Table S2: Tick and Lyme borreliosis risk perceptions, reported overall and stratified by urbanicity, age group, and gender; Table S3: Adoption of tick prevention measures, reported overall and stratified by urbanicity, age group, and gender; Table S4: Likert-scale responses revealing factors influencing willingness to accept Lyme borreliosis vaccination, reported overall and stratified by urbanicity, age group, and gender.

## Abbreviations

The following abbreviations are used in this manuscript:

CI	Confidence interval
DEET	N,N-Diethyl-meta-toluamide
ELISA	Enzyme-linked immunosorbent assay
HCP	Healthcare professional
LB	Lyme borreliosis
SPF	Santé Publique France
