# Supplementary material for: Lyme Borreliosis Awareness, Risk Perception, and Willingness to Vaccinate in France: Where Do We Stand?
Source: Pathogens. 2026 Oct 2;15(10):1050. doi: 10.3390/pathogens15101050 (PMC13708072; doi:10.3390/pathogens15101050)
Supplement: Supplementary file 1 [file pathogens-15-01050-s001.zip › pathogens-4513548-supplementary.pdf]

Supplementary Material for:

"Lyme borreliosis awareness, risk perception, and willingness to vaccinate in France: Where do we stand?"

Table S1. Awareness of ticks and Lyme borreliosis, and past experience of tick bites and Lyme borreliosis reported overall and stratified by urbanicity, age group, and gender.

Characteristic	Aware of ticks % (95% CI)	Have previously been bitten by a tick % (95% CI)*	Aware of Lyme borreliosis % (95% CI)*	Have previously received a Lyme borreliosis diagnosis % (95% CI)$
Overall	94.1 (92.9–95.1)	41.0 (38.7–43.4)	79.2 (77.2–81.1)	7.6 (6.3–9.1)
Urbanicity				
Urban	91.7 (89.5–93.4)	38.6 (35.1–42.2)	72.7 (69.3–75.9)	12.9 (10.3–16.1)
Suburban	92.1 (88.9–94.4)	35.1 (30.3–40.3)	79.9 (75.3–83.8)	5.7 (3.5–9.2)
Rural	98.4 (97.0–99.1)	47.3 (43.4–51.4)	86.6 (83.5–89.1)	3.2 (2.0–5.2)
Age group, years				
18–29	87.2 (83.6–90.1)	40.6 (35.6–45.8)	65.3 (60.0–70.2)	10.4 (7.0–15.2)
30–39	94.2 (91.4–96.2)	45.0 (39.9–50.2)	75.8 (71.1–80.0)	12.3 (8.9–16.8)
40–49	96.6 (94.3–98.0)	42.9 (38.1–47.9)	82.8 (78.7–86.3)	10.4 (7.5–14.3)
50–65	97.0 (95.3–98.1)	37.7 (33.9–41.8)	87.2 (84.1–89.7)	2.2 (1.2–4.0)
Gender				
Women	95.1 (93.4–96.3)	40.2 (37.0–43.6)	84.5 (81.9–86.8)	6.1 (4.6–8.2)
Men	93.1 (91.2–94.6)	41.9 (38.6–45.3)	73.6 (70.4–76.5)	9.2 (7.1–11.8)

* Measured for respondents who reported awareness of ticks.

$ Measured for respondents who reported awareness of ticks and Lyme borreliosis.

Table S2. Tick and Lyme borreliosis risk perceptions, reported overall and stratified by urbanicity, age group, and gender.

		1 (Very low)	2	3	4	5 (Very high)
**Perceived risk of being bitten by a tick, where 1 is "very low risk" and 5 is "very high risk", % (95% CI)***						
Overall	-	10.2 (8.9–11.8)	23.9 (21.9–26.0)	26.5 (24.4–28.6)	30.3 (28.1–32.5)	9.1 (7.8–10.6)
Urbanicity	Urban	12.3 (10.1–14.9)	27.4 (24.3–30.8)	23.0 (20.1–26.2)	27.9 (24.8–31.3)	9.3 (7.4–11.6)
	Suburban	12.5 (9.4–16.4)	23.9 (19.7–28.7)	26.7 (22.3–31.6)	29.7 (25.1–34.7)	7.2 (4.9–10.4)
	Rural	6.4 (4.7–8.6)	19.6 (16.6–23.0)	30.5 (27.0–34.3)	33.5 (29.8–37.4)	10.0 (7.8–12.7)
Age group, years	18–29	13.7 (10.5–17.7)	31.3 (26.7–36.3)	23.7 (19.6–28.4)	24.5 (20.3–29.3)	6.7 (4.5–9.9)
	30–39	8.6 (6.1–12.0)	20.9 (17.0–25.5)	23.3 (19.2–28.0)	33.5 (28.8–38.6)	13.6 (10.4–17.5)
	40–49	8.5 (6.2–11.7)	22.1 (18.3–26.5)	25.8 (21.7–30.4)	34.6 (30.1–39.5)	8.9 (6.5–12.2)
	50–65	10.1 (7.9–12.8)	22.3 (19.1–25.9)	30.4 (26.7–34.2)	29.2 (25.6–33.0)	8.1 (6.1–10.6)
Gender	Women	10.7 (8.8–13.0)	22.1 (19.5–25.0)	25.6 (22.7–28.6)	29.8 (26.9–33.0)	11.8 (9.8–14.1)
	Men	9.7 (7.9–12.0)	25.9 (23.0–29.0)	27.3 (24.3–30.4)	30.8 (27.7–34.0)	6.3 (4.8–8.2)
**Perceived risk of contracting Lyme borreliosis, where 1 is "very low risk" and 5 is "very high risk", % (95% CI)$**						
Overall	-	11.7 (10.1–13.6)	27.1 (24.7–29.6)	31.5 (29.0–34.1)	23.0 (20.8–25.4)	6.7 (5.5–8.2)
Urbanicity	Urban	14.4 (11.6–17.8)	27.2 (23.5–31.3)	26.4 (22.8–30.5)	25.6 (22.0–29.5)	6.3 (4.5–8.8)
	Suburban	13.5 (9.9–18.1)	27.5 (22.5–33.2)	33.3 (27.9–39.1)	20.5 (16.1–25.7)	5.2 (3.1–8.7)
	Rural	8.0 (5.9–10.8)	26.7 (23.1–30.8)	35.5 (31.5–39.8)	21.8 (18.4–25.6)	7.9 (5.8–10.6)
Age group, years	18–29	13.5 (9.6–18.7)	30.5 (24.7–37.0)	30.4 (24.6–36.9)	20.6 (15.8–26.5)	5.0 (2.8–8.8)
	30–39	8.8 (5.9–12.9)	21.7 (17.1–27.1)	30.9 (25.6–36.7)	28.5 (23.4–34.3)	10.1 (7.0–14.4)
	40–49	9.6 (6.8–13.4)	25.3 (20.7–30.4)	28.9 (24.1–34.2)	28.6 (23.8–33.8)	7.7 (5.2–11.2)
	50–65	13.7 (10.9–17.0)	29.4 (25.6–33.6)	33.8 (29.7–38.1)	17.9 (14.8–21.6)	5.2 (3.5–7.5)
Gender	Women	11.6 (9.5–14.2)	26.3 (23.2–29.7)	33.6 (30.2–37.2)	20.3 (17.5–23.5)	8.1 (6.3–10.4)
	Men	11.9 (9.5–14.8)	28.2 (24.6–32.0)	28.7 (25.2–32.6)	26.4 (23.0–30.2)	4.8 (3.4–6.9)
**Perceived severity of Lyme borreliosis, where 1 is "not severe at all" and 5 is "extremely severe", % (95% CI)$**						
Overall	-	0.6 (0.3–1.2)	2.6 (1.9–3.7)	13.6 (11.8–15.6)	56.1 (53.4–58.8)	27.1 (24.7–29.6)
Urbanicity	Urban	1.0 (0.4–2.4)	2.3 (1.3–4.1)	14.7 (11.9–18.1)	57.1 (52.8–61.4)	24.8 (21.3–28.8)
	Suburban	0.3 (0.0–2.5)	2.9 (1.5–5.8)	13.5 (9.9–18.1)	55.8 (49.8–61.6)	27.4 (22.4–33.1)
	Rural	0.2 (0.0–1.5)	2.8 (1.6–4.6)	12.6 (10.0–15.8)	55.2 (50.8–59.5)	29.2 (25.4–33.4)
Age group, years	18–29	0.9 (0.2–3.7)	3.7 (1.8–7.2)	15.4 (11.2–20.9)	57.5 (50.8–63.9)	22.5 (17.4–28.5)
	30–39	0.4 (0.0–2.5)	4.2 (2.3–7.4)	15.9 (11.9–20.8)	55.5 (49.4–61.4)	24.1 (19.3–29.7)
	40–49	0.3 (0.0–2.2)	1.6 (0.7–3.8)	13.3 (9.9–17.5)	58.7 (53.1–64.0)	26.1 (21.6–31.3)
	50–65	0.6 (0.2–2.0)	2.0 (1.1–3.7)	11.8 (9.3–15.0)	54.3 (49.8–58.6)	31.2 (27.3–35.5)
Gender	Women	0.6 (0.2–1.6)	2.0 (1.2–3.4)	13.4 (11.1–16.1)	53.0 (49.3–56.7)	31.0 (27.7–34.6)
	Men	0.5 (0.2–1.6)	3.4 (2.2–5.2)	14.0 (11.4–17.0)	60.0 (56.0–64.0)	22.1 (18.9–25.7)
**Concern about contracting Lyme borreliosis, where 1 is "not at all concerned" and 5 is "extremely concerned", % (95% CI)$**						
Overall	-	8.9 (7.4–10.5)	20.7 (18.6–23.0)	24.4 (22.1–26.8)	30.9 (28.5–33.5)	15.1 (13.2–17.2)
Urbanicity	Urban	9.7 (7.4–12.6)	21.5 (18.2–25.3)	23.1 (19.7–27.0)	29.8 (26.0–33.9)	15.9 (13.0–19.3)
	Suburban	9.9 (6.8–14.0)	19.9 (15.5–25.1)	26.0 (21.1–31.6)	33.9 (28.5–39.8)	10.4 (7.2–14.6)
	Rural	7.5 (5.5–10.1)	20.3 (17.0–24.1)	24.9 (21.3–28.8)	30.5 (26.6–34.7)	16.8 (13.8–20.4)
Age group, years	18–29	7.6 (4.8–12.0)	20.9 (16.0–26.9)	24.2 (19.0–30.4)	31.6 (25.8–38.1)	15.6 (11.3–21.1)
	30–39	7.2 (4.6–11.0)	18.4 (14.2–23.6)	22.5 (17.9–28.0)	33.0 (27.6–39.0)	18.8 (14.6–24.0)
	40–49	8.6 (5.9–12.2)	18.6 (14.7–23.4)	22.3 (18.0–27.2)	33.0 (28.0–38.4)	17.5 (13.7–22.2)
	50–65	10.4 (8.0–13.5)	23.0 (19.4–26.9)	26.7 (23.0–30.8)	28.3 (24.5–32.5)	11.5 (9.0–14.7)
Gender	Women	7.8 (6.0–10.0)	20.5 (17.7–23.6)	24.8 (21.8–28.2)	29.0 (25.8–32.5)	17.8 (15.2–20.8)
	Men	10.2 (8.0–13.0)	20.9 (17.8–24.4)	24.0 (20.7–27.7)	33.4 (29.7–37.4)	11.5 (9.1–14.3)

* Measured for respondents who reported awareness of ticks.

$ Measured for respondents who reported awareness of ticks and Lyme borreliosis.

Table S3. Adoption of tick prevention measures, reported overall and stratified by urbanicity, age group, and gender.*

		Never	Seldom	Sometimes	Often	Always
**Conducting tick checks, % (95% CI)**						
Overall	-	20.0 (18.1–22.0)	20.5 (18.6–22.4)	14.2 (12.6–15.9)	25.6 (23.5–27.7)	14.2 (12.6–16.0)
Urbanicity	Urban	21.7 (18.9–24.9)	23.4 (20.5–26.6)	12.8 (10.5–15.4)	24.2 (21.3–27.5)	11.5 (9.4–14.0)
	Suburban	23.9 (19.7–28.6)	21.5 (17.5–26.2)	14.3 (11.0–18.4)	24.2 (20.0–29.0)	10.8 (7.9–14.5)
	Rural	15.6 (12.9–18.8)	16.2 (13.5–19.4)	15.8 (13.1–19.0)	27.9 (24.5–31.7)	19.5 (16.5–22.9)
Age group, years	18–29	15.7 (12.3–19.9)	22.6 (18.6–27.2)	14.8 (11.5–18.9)	27.1 (22.7–31.9)	14.5 (11.2–18.6)
	30–39	18.6 (14.9–23.0)	20.0 (16.2–24.5)	13.9 (10.7–17.9)	30.5 (26.0–35.4)	13.4 (10.3–17.4)
	40–49	17.9 (14.4–22.0)	20.0 (16.3–24.2)	15.3 (12.1–19.2)	23.9 (19.9–28.4)	15.7 (12.4–19.6)
	50–65	24.7 (21.4–28.4)	19.7 (16.7–23.1)	13.2 (10.7–16.3)	22.8 (19.5–26.4)	13.6 (11.0–16.7)
Gender	Women	19.7 (17.2–22.5)	18.1 (15.7–20.8)	14.2 (12.0–16.7)	25.3 (22.5–28.3)	18.3 (15.9–21.1)
	Men	20.2 (17.6–23.1)	22.9 (20.1–25.9)	14.2 (12.0–16.8)	25.9 (23.0–29.0)	9.7 (7.9–11.9)
**Using protective clothing, % (95% CI)**						
Overall	-	18.6 (16.8–20.6)	23.4 (21.4–25.4)	14.9 (13.3–16.7)	26.7 (24.6–28.9)	11.8 (10.3–13.4)
Urbanicity	Urban	19.3 (16.6–22.3)	23.6 (20.7–26.8)	15.4 (13.0–18.2)	26.0 (22.9–29.2)	10.0 (8.1–12.4)
	Suburban	19.9 (16.1–24.5)	25.2 (20.9–30.0)	13.5 (10.3–17.5)	27.1 (22.7–32.0)	10.9 (8.0–14.7)
	Rural	17.1 (14.3–20.3)	22.0 (18.9–25.5)	15.1 (12.5–18.2)	27.4 (23.9–31.1)	14.4 (11.8–17.4)
Age group, years	18–29	20.1 (16.2–24.6)	22.9 (18.8–27.5)	12.3 (9.3–16.2)	27.7 (23.3–32.6)	11.1 (8.2–14.8)
	30–39	17.9 (14.3–22.2)	24.3 (20.1–29.0)	17.4 (13.8–21.6)	26.1 (21.8–30.8)	11.6 (8.7–15.4)
	40–49	17.8 (14.3–21.9)	25.0 (21.0–29.5)	15.4 (12.1–19.3)	25.5 (21.4–30.1)	10.6 (7.9–14.1)
	50–65	18.7 (15.7–22.1)	22.1 (18.9–25.7)	14.7 (12.1–17.9)	27.2 (23.7–31.0)	13.0 (10.5–16.0)
Gender	Women	20.0 (17.5–22.9)	22.1 (19.5–25.0)	14.0 (11.8–16.5)	26.9 (24.0–29.9)	13.4 (11.2–15.8)
	Men	17.2 (14.8–20.0)	24.5 (21.7–27.6)	15.9 (13.6–18.6)	26.4 (23.5–29.5)	10.2 (8.3–12.4)
**Avoiding tick-infested areas, % (95% CI)**						
Overall	-	13.4 (11.8–15.1)	24.1 (22.1–26.2)	17.0 (15.2–18.8)	24.3 (22.4–26.5)	11.3 (9.9–12.9)
Urbanicity	Urban	14.0 (11.7–16.7)	24.6 (21.6–27.8)	17.1 (14.6–20.0)	22.1 (19.3–25.2)	11.8 (9.6–14.3)
	Suburban	14.2 (10.9–18.2)	21.5 (17.5–26.2)	18.6 (14.8–23.1)	20.4 (16.5–24.9)	14.1 (10.8–18.2)
	Rural	12.1 (9.7–15.0)	24.9 (21.6–28.5)	15.9 (13.1–19.0)	29.3 (25.8–33.1)	9.2 (7.1–11.8)
Age group, years	18–29	10.6 (7.8–14.2)	26.6 (22.3–31.4)	16.7 (13.2–20.9)	26.1 (21.8–30.9)	12.0 (9.0–15.8)
	30–39	14.5 (11.3–18.6)	21.7 (17.7–26.2)	15.3 (11.9–19.4)	25.9 (21.6–30.7)	15.2 (11.8–19.3)
	40–49	13.9 (10.8–17.7)	28.4 (24.1–33.0)	15.5 (12.2–19.4)	23.7 (19.8–28.2)	10.5 (7.8–13.9)
	50–65	14.0 (11.4–17.1)	21.3 (18.2–24.8)	19.0 (16.0–22.5)	22.8 (19.5–26.4)	9.2 (7.1–11.8)
Gender	Women	15.0 (12.7–17.5)	22.0 (19.4–24.9)	16.1 (13.7–18.7)	25.8 (23.0–28.9)	13.3 (11.2–15.8)
	Men	11.8 (9.7–14.1)	26.4 (23.5–29.5)	18.0 (15.5–20.8)	22.7 (19.9–25.6)	9.0 (7.2–11.2)
**Using insect repellents, % (95% CI)**						
Overall	-	43.1 (40.7–45.5)	20.5 (18.6–22.5)	9.9 (8.6–11.4)	11.8 (10.4–13.5)	6.1 (5.1–7.4)
Urbanicity	Urban	37.8 (34.4–41.4)	22.1 (19.3–25.2)	11.0 (8.9–13.4)	13.9 (11.6–16.6)	6.8 (5.2–8.9)
	Suburban	49.3 (44.0–54.5)	20.4 (16.5–25.0)	7.2 (4.9–10.4)	8.7 (6.1–12.1)	5.4 (3.5–8.4)
	Rural	46.0 (42.0–50.0)	18.6 (15.7–21.9)	10.2 (8.0–12.9)	11.2 (8.9–14.0)	5.6 (4.0–7.8)
Age group, years	18–29	33.0 (28.3–38.1)	23.2 (19.1–27.9)	11.6 (8.6–15.4)	14.8 (11.5–18.9)	8.4 (5.9–11.8)
	30–39	35.4 (30.6–40.5)	22.5 (18.5–27.1)	12.9 (9.8–16.8)	16.6 (13.1–20.8)	6.7 (4.5–9.8)
	40–49	41.5 (36.7–46.4)	23.5 (19.6–28.0)	8.2 (5.8–11.3)	11.4 (8.6–15.0)	6.9 (4.7–9.8)
	50–65	54.7 (50.6–58.7)	15.8 (13.0–19.0)	8.2 (6.3–10.8)	7.5 (5.6–10.0)	3.9 (2.6–5.8)
Gender	Women	44.9 (41.6–48.3)	19.1 (16.7–21.9)	8.7 (7.0–10.8)	11.9 (9.9–14.2)	7.2 (5.6–9.1)
	Men	41.1 (37.8–44.5)	22.0 (19.3–25.0)	11.1 (9.1–13.4)	11.9 (9.8–14.3)	4.9 (3.6–6.6)

* Measured for respondents who reported awareness of ticks. The proportion of respondents answering "Unknown" is not shown.

Table S4. Likert-scale responses revealing factors influencing willingness to accept Lyme borreliosis vaccination, reported overall and stratified by urbanicity, age group, and gender.*

		**1 (Strongly disagree)**	**2**	**3**	**4**	**5 (Strongly agree)**
**Workplace offers vaccination, % (95% CI)**						
Overall	-	19.0 (16.9–21.3)	16.6 (14.7–18.7)	24.4 (22.2–26.9)	25.3 (23.0–27.7)	14.7 (12.9–16.8)
Urbanicity	Urban	13.6 (10.9–16.9)	14.1 (11.3–17.4)	24.7 (21.1–28.7)	31.4 (27.5–35.5)	16.2 (13.2–19.6)
	Suburban	19.7 (15.4–24.9)	20.0 (15.6–25.1)	24.0 (19.3–29.5)	24.1 (19.4–29.6)	12.2 (8.8–16.7)
	Rural	24.0 (20.5–27.9)	17.3 (14.3–20.9)	24.4 (20.8–28.3)	19.8 (16.5–23.5)	14.6 (11.7–18.0)
Age group, years	18-29	12.8 (9.0–18.0)	20.0 (15.2–25.9)	24.4 (19.2–30.6)	29.0 (23.3–35.3)	13.7 (9.7–19.0)
	30-39	16.7 (12.7–21.8)	15.0 (11.2–19.9)	20.4 (15.9–25.7)	33.3 (27.8–39.2)	14.6 (10.8–19.4)
	40-49	17.7 (13.8–22.4)	19.4 (15.3–24.1)	23.4 (19.0–28.4)	26.2 (21.6–31.4)	13.3 (10.0–17.6)
	50–65	23.6 (20.1–27.6)	14.3 (11.4–17.7)	27.1 (23.3–31.3)	19.0 (15.7–22.7)	16.0 (13.0–19.6)
Gender	Women	21.7 (18.8–25.0)	19.5 (16.7–22.6)	23.2 (20.2–26.4)	22.4 (19.4–25.6)	13.2 (10.9–15.9)
	Men	15.5 (12.8–18.8)	13.1 (10.6–16.1)	25.9 (22.5–29.7)	28.9 (25.4–32.8)	16.4 (13.6–19.7)
**Healthcare professional recommendation, % (95% CI)**						
Overall	-	8.2 (6.8–9.8)	9.2 (7.7–10.9)	19.3 (17.2–21.5)	36.1 (33.5–38.8)	27.2 (24.9–29.7)
Urbanicity	Urban	6.3 (4.4–8.8)	9.1 (6.9–12.0)	18.3 (15.2–22.0)	38.5 (34.4–42.8)	27.7 (24.0–31.8)
	Suburban	8.8 (6.0–12.9)	9.9 (6.9–14.1)	19.7 (15.4–24.8)	35.2 (29.7–41.1)	26.4 (21.4–31.9)
	Rural	9.7 (7.4–12.6)	8.8 (6.6–11.6)	20.0 (16.7–23.7)	34.2 (30.2–38.5)	27.3 (23.5–31.3)
Age group, years	18–29	5.5 (3.1–9.4)	9.0 (5.9–13.6)	23.2 (18.1–29.3)	38.1 (31.9–44.7)	24.2 (19.0–30.4)
	30–39	7.7 (5.0–11.6)	10.1 (7.0–14.4)	15.3 (11.5–20.2)	40.5 (34.7–46.6)	26.4 (21.4–32.0)
	40–49	7.1 (4.7–10.6)	10.9 (7.9–14.9)	21.8 (17.5–26.7)	35.5 (30.4–41.0)	24.7 (20.2–29.8)
	50–65	10.3 (7.8–13.3)	7.8 (5.7–10.5)	18.1 (14.9–21.7)	33.4 (29.3–37.7)	30.5 (26.6–34.8)
Gender	Women	9.4 (7.5–11.8)	9.5 (7.6–11.9)	20.4 (17.5–23.5)	33.5 (30.1–37.1)	27.2 (24.0–30.6)
	Men	6.6 (4.9–9.0)	8.8 (6.7–11.4)	17.7 (14.8–21.0)	39.5 (35.6–43.6)	27.3 (23.8–31.1)
**Official guideline publication, % (95% CI)**						
Overall	-	9.3 (7.8–11.1)	13.4 (11.6–15.4)	25.0 (22.7–27.5)	34.0 (31.5–36.7)	18.2 (16.2–20.5)
Urbanicity	Urban	6.6 (4.7–9.2)	13.3 (10.6–16.5)	21.9 (18.5–25.8)	37.6 (33.5–41.9)	20.6 (17.3–24.3)
	Suburban	8.2 (5.4–12.1)	16.1 (12.2–21.0)	25.8 (20.9–31.4)	35.7 (30.2–41.6)	14.2 (10.6–18.9)
	Rural	12.7 (10.0–15.9)	12.1 (9.5–15.2)	27.7 (23.9–31.7)	29.5 (25.7–33.7)	18.0 (14.9–21.7)
Age group, years	18–29	6.4 (3.8–10.6)	15.6 (11.4–21.1)	22.0 (17.0–28.0)	35.4 (29.3–42.0)	20.6 (15.7–26.5)
	30–39	8.7 (5.8–12.8)	15.4 (11.5–20.3)	21.3 (16.7–26.6)	36.9 (31.2–42.9)	17.8 (13.6–22.9)
	40–49	8.4 (5.8–12.1)	16.4 (12.7–20.9)	25.4 (20.9–30.6)	36.4 (31.2–41.8)	13.5 (10.1–17.8)
	50–65	11.5 (8.9–14.6)	9.6 (7.3–12.6)	28.0 (24.2–32.2)	30.6 (26.6–34.8)	20.3 (16.9–24.1)
Gender	Women	11.1 (9.0–13.6)	14.4 (12.0–17.2)	23.7 (20.7–27.0)	33.3 (29.9–36.9)	17.5 (14.8–20.5)
	Men	7.2 (5.3–9.7)	12.1 (9.7–15.0)	26.6 (23.1–30.3)	35.0 (31.2–39.0)	19.1 (16.1–22.5)
**Friend and family recommendation, % (95% CI)**						
Overall	-	11.4 (9.8–13.3)	14.8 (13.0–16.9)	28.7 (26.2–31.2)	30.7 (28.3–33.3)	14.4 (12.6–16.4)
Urbanicity	Urban	8.7 (6.5–11.5)	13.3 (10.6–16.5)	25.4 (21.8–29.3)	35.4 (31.3–39.6)	17.3 (14.2–20.8)
	Suburban	12.5 (9.1–17.0)	14.9 (11.1–19.6)	29.5 (24.4–35.2)	30.1 (25.0–35.9)	13.0 (9.4–17.6)
	Rural	13.5 (10.8–16.8)	16.3 (13.3–19.8)	31.5 (27.6–35.7)	26.5 (22.8–30.5)	12.2 (9.6–15.4)
Age group, years	18–29	5.5 (3.1–9.4)	13.2 (9.3–18.4)	23.6 (18.4–29.6)	42.6 (36.2–49.3)	15.1 (10.9–20.6)
	30–39	10.4 (7.2–14.8)	14.2 (10.5–18.9)	23.9 (19.1–29.4)	33.6 (28.2–39.5)	17.9 (13.7–23.1)
	40–49	11.9 (8.7–16.0)	18.5 (14.6–23.2)	28.1 (23.4–33.4)	29.7 (24.9–35.0)	11.8 (8.6–15.9)
	50–65	14.3 (11.5–17.7)	13.7 (10.9–17.0)	33.6 (29.6–38.0)	24.6 (21.0–28.6)	13.8 (11.0–17.1)
Gender	Women	12.2 (10.0–14.9)	16.7 (14.2–19.7)	28.7 (25.4–32.1)	28.7 (25.5–32.2)	13.7 (11.3–16.4)
	Men	10.5 (8.2–13.3)	12.3 (9.9–15.3)	28.6 (25.1–32.5)	33.4 (29.7–37.4)	15.1 (12.4–18.3)
**Social media, % (95% CI)**						
Overall	-	30.5 (28.0–33.1)	21.1 (18.9–23.4)	23.7 (21.4–26.1)	18.1 (16.1–20.3)	6.7 (5.5–8.2)
Urbanicity	Urban	23.3 (19.8–27.2)	19.7 (16.5–23.4)	22.9 (19.5–26.8)	25.1 (21.6–29.0)	8.9 (6.7–11.7)
	Suburban	32.7 (27.3–38.5)	22.4 (17.8–27.8)	25.4 (20.6–31.0)	13.1 (9.6–17.7)	6.3 (4.0–10.0)
	Rural	36.4 (32.3–40.8)	21.7 (18.3–25.5)	23.5 (20.0–27.4)	13.7 (10.9–17.0)	4.7 (3.2–6.9)
Age group, years	18–29	15.9 (11.6–21.4)	27.9 (22.4–34.3)	20.3 (15.5–26.2)	27.3 (21.8–33.6)	8.6 (5.5–13.1)
	30–39	27.3 (22.2–33.0)	19.4 (15.1–24.7)	19.0 (14.7–24.2)	22.9 (18.3–28.4)	11.3 (8.0–15.7)
	40–49	27.9 (23.2–33.1)	21.9 (17.7–26.8)	23.6 (19.2–28.6)	20.3 (16.2–25.1)	6.3 (4.1–9.6)
	50–65	40.1 (35.8–44.5)	18.3 (15.1–22.0)	27.7 (23.9–31.8)	10.2 (7.8–13.2)	3.7 (2.4–5.8)
Gender	Women	32.7 (29.3–36.3)	22.8 (19.9–26.1)	22.2 (19.2–25.4)	15.2 (12.7–18.0)	7.1 (5.5–9.3)

		1 (Strongly disagree)	2	3	4	5 (Strongly agree)
	Men	27.9 (24.4–31.7)	18.8 (15.8–22.2)	25.5 (22.1–29.3)	21.7 (18.6–25.3)	6.0 (4.3–8.3)
**Cost, % (95% CI)**						
Overall	-	13.1 (11.4–15.1)	19.6 (17.5–21.9)	24.8 (22.6–27.3)	24.1 (21.8–26.5)	18.4 (16.3–20.6)
Urbanicity	Urban	11.1 (8.6–14.2)	17.2 (14.1–20.7)	23.1 (19.7–27.0)	27.1 (23.4–31.1)	21.6 (18.2–25.4)
	Suburban	14.0 (10.3–18.6)	25.1 (20.3–30.6)	25.1 (20.3–30.7)	20.8 (16.4–26.0)	15.0 (11.2–19.8)
	Rural	14.7 (11.9–18.1)	19.2 (16.0–22.8)	26.4 (22.7–30.4)	22.8 (19.3–26.7)	17.0 (13.9–20.5)
Age group, years	18–29	6.3 (3.8–10.4)	22.0 (16.9–28.0)	23.2 (18.0–29.2)	27.1 (21.6–33.4)	21.5 (16.5–27.4)
	30–39	10.3 (7.1–14.6)	18.9 (14.6–24.1)	21.7 (17.1–27.1)	28.9 (23.7–34.6)	20.3 (15.8–25.6)
	40–49	11.0 (7.9–15.0)	16.8 (13.0–21.3)	27.3 (22.6–32.5)	29.4 (24.6–34.7)	15.6 (12.0–20.0)
	50–65	18.8 (15.6–22.6)	20.7 (17.3–24.5)	25.7 (22.0–29.8)	17.1 (14.0–20.7)	17.7 (14.6–21.3)
Gender	Women	13.2 (10.9–16.0)	20.2 (17.4–23.3)	25.0 (22.0–28.3)	21.1 (18.3–24.3)	20.4 (17.6–23.6)
	Men	13.0 (10.5–16.0)	18.9 (15.9–22.3)	24.6 (21.2–28.3)	27.8 (24.3–31.6)	15.8 (13.0–19.0)
**Fear of side effects, % (95% CI)**						
Overall	-	6.4 (5.2–7.9)	12.3 (10.6–14.2)	18.1 (16.1–20.3)	32.1 (29.6–34.7)	31.1 (28.6–33.7)
Urbanicity	Urban	7.6 (5.6–10.3)	11.9 (9.4–15.1)	15.9 (13.0–19.3)	35.4 (31.4–39.7)	29.1 (25.3–33.2)
	Suburban	6.6 (4.2–10.2)	13.7 (10.1–18.3)	18.5 (14.3–23.6)	30.7 (25.5–36.5)	30.6 (25.4–36.3)
	Rural	5.1 (3.4–7.4)	11.9 (9.3–15.1)	20.1 (16.9–23.9)	29.6 (25.7–33.7)	33.3 (29.3–37.6)
Age group, years	18–29	6.1 (3.6–10.3)	15.7 (11.5–21.2)	15.8 (11.5–21.2)	34.2 (28.2–40.7)	28.2 (22.6–34.6)
	30–39	5.2 (3.1–8.6)	8.9 (6.0–12.9)	18.4 (14.1–23.5)	30.6 (25.3–36.4)	37.0 (31.4–43.0)
	40–49	4.8 (2.9–7.9)	11.6 (8.5–15.7)	17.0 (13.3–21.6)	33.4 (28.4–38.9)	33.1 (28.1–38.5)
	50–65	8.1 (6.0–10.8)	12.9 (10.2–16.2)	19.7 (16.4–23.5)	31.2 (27.2–35.4)	28.2 (24.3–32.3)
Gender	Women	5.1 (3.7–7.0)	9.5 (7.5–11.9)	15.9 (13.3–18.7)	31.0 (27.7–34.5)	38.5 (35.0–42.2)
	Men	7.9 (5.9–10.4)	15.8 (13.1–19.0)	20.8 (17.7–24.3)	33.4 (29.7–37.4)	22.1 (18.9–25.6)
**Multidose vaccine, % (95% CI)**						
Overall	-	10.0 (8.4–11.7)	12.0 (10.3–13.9)	24.4 (22.1–26.8)	30.2 (27.7–32.8)	23.5 (21.2–25.9)
Urbanicity	Urban	9.2 (6.9–12.0)	11.8 (9.2–14.9)	22.6 (19.2–26.5)	32.1 (28.1–36.2)	24.4 (20.9–28.3)
	Suburban	11.7 (8.4–16.2)	12.9 (9.4–17.5)	21.4 (16.9–26.7)	29.6 (24.5–35.4)	24.3 (19.5–29.8)
	Rural	9.8 (7.5–12.8)	11.6 (9.1–14.7)	27.8 (24.1–31.9)	28.6 (24.8–32.7)	22.1 (18.7–25.9)
Age group, years	18–29	6.8 (4.1–11.1)	14.7 (10.6–20.2)	25.8 (20.4–32.0)	33.6 (27.7–40.2)	19.0 (14.3–24.8)
	30–39	8.8 (5.9–12.9)	9.1 (6.2–13.3)	17.9 (13.8–23.0)	38.3 (32.6–44.4)	25.8 (20.9–31.4)
	40–49	12.2 (9.0–16.3)	14.0 (10.6–18.3)	27.4 (22.7–32.6)	27.1 (22.4–32.3)	19.4 (15.4–24.1)
	50–65	10.7 (8.2–13.7)	11.0 (8.5–14.1)	25.4 (21.7–29.4)	26.3 (22.6–30.4)	26.6 (22.9–30.7)
Gender	Women	12.3 (10.1–14.9)	13.4 (11.1–16.2)	23.6 (20.6–26.9)	27.4 (24.2–30.8)	23.3 (20.3–26.6)
	Men	7.2 (5.3–9.6)	10.2 (8.0–13.0)	25.4 (22.0–29.1)	33.7 (29.9–37.6)	23.6 (20.3–27.2)

* Measured for respondents who reported awareness of ticks and Lyme borreliosis.
